# Visit-to-visit variability of serum uric acid measurements and the risk of all-cause mortality in the general population

**DOI:** 10.1186/s13075-021-02445-7

**Published:** 2021-03-04

**Authors:** Xue Tian, Anxin Wang, Yingting Zuo, Shuohua Chen, Licheng Zhang, Shouling Wu, Yanxia Luo

**Affiliations:** 1 Department of Epidemiology and Health Statistics, School of Public Health, Capital Medical University, No.10 Xitoutiao, You’anmen Wai, Fengtai District, Beijing, 100069 China; 2 Beijing Municipal Key Laboratory of Clinical Epidemiology, Beijing, China; 3 China National Clinical Research Center for Neurological Diseases, Beijing Tiantan Hospital, Capital Medical University, Beijing, China; 4 Department of Neurology, Beijing Tiantan Hospital, Capital Medical University, Beijing, China; 5 Department of Cardiology, Kailuan Hospital, North China University of Science and Technology, 57 Xinhua East Rd, Tangshan, 063000 China

**Keywords:** Serum uric acid, Variability, All-cause mortality, Risk factors

## Abstract

**Background:**

Evidence on longitudinal variability of serum uric acid (SUA) and risk of all-cause mortality in the general population is limited, as many prior studies focused on a single measurement of SUA.

**Methods:**

A total of 53,956 participants in the Kailuan study who underwent three health examinations during 2006 to 2010 were enrolled. Variability of SUA was measured using the coefficient of variation (primary index), standard deviation, average real variability, and variability independent of the mean. Cox proportional hazard regressions were used to calculate the hazard ratio (HR) and 95% confidence interval (CI) for the association of variability of SUA with subsequent risk of all-cause mortality, considering its magnitude and the direction and across different baseline SUA categories.

**Results:**

Over a median follow-up of 7.04 years, 2728 participants died. The highest variability of SUA was associated with an increased risk of all-cause mortality, the HR was 1.33 (95% CI, 1.20–1.49) compared with the lowest variability. In this group, both a large fall (HR, 1.28; 95% CI, 1.14–1.44) and rise (HR, 1.18; 95% 1.05–1.32) in SUA were related to risk of all-cause mortality. These associations were similar across different baseline SUA categories. Consistent results were observed in alternative measures of SUA variability. Moreover, individuals with higher variability in SUA were more related to common risk factors than those with stable SUA.

**Conclusions:**

Higher variability in SUA was independently associated with the risk of all-cause mortality irrespective of baseline SUA and direction of variability in the general population.

## Background

Serum uric acid (SUA) is the final enzymatic product of purine metabolism, and accumulation of SUA is well known to cause monosodium urate crystal deposition in the joints and kidneys, leading to the development of gout and kidney stones [1]. Experimental and epidemiologic studies suggest that elevated levels of SUA are associated with a wide variety of adverse health outcomes, including hypertension [2], obesity [3], diabetes mellitus [4], dyslipidemia [5], renal disorder [6], and cardiovascular and cerebrovascular events [7], which may reduce the longevity of the affected individuals.

Moreover, SUA has been considered a risk factor for mortality in some previous studies [8–14]; however, these associations were controversial and only based on a single baseline SUA measurement, which may not reflect chronic SUA exposure and its attendant risk of mortality.

Recently, clinical observations have demonstrated that increased variability of body mass index (BMI), blood pressure, glucose, and cholesterols were associated with increased risk of mortality [15–18]. Nevertheless, studies regarding variability of SUA and outcomes were not fully elucidated. Three previous studies have investigated the effect of variability of SUA (only measured by standard deviation [SD]) on outcomes and revealed that higher variability of SUA was related to future cardiovascular events in patients undergoing percutaneous coronary intervention [19], coronary heart disease in male workers [20], and chronic kidney in type 2 diabetes [21]. While whether various variability measurements in SUA are each associated with risk of all-cause mortality in the general population remained unknown, we also did not know whether this putative association differed by baseline SUA and the fall or rise in SUA. Therefore, the purpose of our study was to evaluate the associations between various variability measurements in SUA over 4 years with risk of all-cause mortality in the general population, considering different baseline SUA categories and the direction of variability.

## Methods

### Study design and participants

The Kailuan study is a prospective cohort study conducted in the Kailuan community in Tangshan city, China. The detailed study design and characteristics of the study have been described previously [22, 23]. In brief, 101,510 (81,110 men and 20,400 women) participants aged 18 to 98 years were enrolled in the community and underwent questionnaire assessments, clinical examinations, and laboratory tests biennially until December 31, 2017 (SFigure 1, see Additional file 1). A total of 56,833 participants underwent 3 follow-ups from 2006 to 2010, and we further excluded 916 participants who died and 2587 participants with missing data regarding serum uric acid levels in or before 2010. Finally, 53,956 participants were included in the present analysis (SFigure 2, see Additional file 2). The study was performed according to the guidelines of the Helsinki Declaration and was approved by the Ethics Committee of Kailuan General Hospital (approval number 2006-05) and Beijing Tiantan Hospital (approval number 2010-014-01). All participants provided informed written consent.

### Measurement of SUA change and variability

Fasting blood samples were collected in the morning after an 8- to 12-h overnight fast and transfused into vacuum tubes containing EDTA. The concentration of SUA was examined with a commercial kit (Ke Hua Biological Engineering Corporation, Shanghai, China) using an automatic biochemical analyzer (Hitachi 7600, Tokyo, Japan) according to the manufacturer’s instructions.

Visit-to-visit SUA variability between three visits (in years 2006, 2008, and 2010) was the exposure of interest in the present study. Indices of visit-to-visit SUA variability include the coefficient of variation (CV), SD, average real variability (ARV), and variability independent of the mean (VIM). The CV was calculated as (SD/mean) × 100% [24]. The ARV calculated as the average absolute difference between successive measurements [25]. The VIM was calculated as 100 × SD/mean^beta^, where beta is the regression coefficient based on a natural logarithm of SD on the natural logarithm of the mean [26]. The CV was presented as the primary exposure variable of interest. We further stratified SUA variability into 4 categories according to quartiles, for the direction of variability of SUA, a large fall and rise of SUA were defined as decreased and increased SUA those with the highest variability (Q4) group, respectively.

### Assessment of potential covariates

Information on demographic, socioeconomic status, medical history, and lifestyle information was collected using a self-reported questionnaire. Educational attainment was categorized as illiteracy or primary school, middle school, and high school or above. Income level was categorized as < 1000 RMB and ≥ 1000 RMB. Physical activity was classified as inactive activity (< 80 per week) and active activity (≥ 80 min of activity per week). Smoking status and alcohol use were classified as never, former, or current according to self-reported information. Weight and height were measured and BMI was calculated as weight (kg)/height (m)^2^. Systolic blood pressure (SBP) and diastolic blood pressure (DBP) were measured 3 times with the participants in the seated position using a mercury sphygmomanometer, and the average of 3 readings was used in the analyses. All blood samples were tested using a Hitachi 747 auto-analyzer (Hitachi, Tokyo, Japan) at the central laboratory of the Kailuan Hospital. Fasting blood glucose was measured with the hexokinase/glucose-6-phosphate dehydrogenase method. Serum creatinine was measured with the sarcosine oxidase assay method. Estimated glomerular filtration rate (eGFR) was calculated using the creatinine-based Chronic Kidney Disease Epidemiological Collaboration equation [27]. Plasma high-sensitivity C-reactive protein (hs-CRP) was measured with high-sensitivity particle-enhanced immunonephelometry assay. Hypertension was defined as any self-reported hypertension or use of antihypertensive drug, or BP ≥ 140/90 mmHg. Diabetes mellitus was defined as any self-reported diabetes mellitus or use of glucose-lowering drugs, or fasting blood glucose ≥ 7 mmol/L. Dyslipidemia was defined as any self-reported history or use of lipid-lowering drugs, or serum total cholesterol (TC) ≥ 5.17 mmol/L or triglyceride ≥ 1.69 mmol/L or low-density lipoprotein cholesterol ≥ 3.62 mmol/L or high-density lipoprotein cholesterol ≤ 1.04 mmol/L.

### Study outcomes and follow-up

The outcome of the study was all-cause death, which was defined as death from any cause and ascertained annually by professional doctors based on examination of death certificates from provincial vital statistics offices. Participants were followed after 2010 via face-to-face interviews at every 2-year routine medical examination until December 31, 2017, or until the time of death, whichever came first.

### Statistical analysis

Continuous variables were presented as mean ± SD and compared with Student’s *t* test or ANOVA. Categorical variables were reported as frequencies (percentages) and compared using chi-square test. The incidence rate of all-cause mortality was calculated by dividing the number of incident case by total follow-up duration (per 1000 person-years). We analyzed the effect of baseline SUA on all-cause mortality as a continuous variable using restricted cubic splines with 3 knots (10th, 50th, 90th percentile) [28] and adjusted for the same variables as in the Cox regression analyses (see below). Test of linear and nonlinear terms were based on the likelihood ratio test. Survival analysis was performed by the Kaplan-Meier method and log-rank test.

Separate multivariable Cox proportional hazards models were used to evaluate the association of categorical and continuous measures of variability of SUA with all-cause mortality in the overall cohort and across subpopulation stratified by baseline SUA (< 300 and ≥ 300 μmol/L). Three models were constructed, model 1 was adjusted for age and gender; model 2 was further adjusted for BMI, SBP, DBP, FBG, education, income, smoking status, drinking status, physical activity, history of hypertension, diabetes and dyslipidemia, baseline SUA, and mean SUA; and model 3 was further adjusted for antihypertensive agents, hypoglycemic agents, lipid-lowering agents, eGFR, and hs-CRP. Multivariable Cox proportional regression models were used to analyze the relationship of a large fall or rise in SUA with all-cause mortality.

Additional analyses were performed to evaluate the robustness of the association of variability in SUA and all-cause mortality. First, to account for the potential contribution of hyperuricemia events (defined as SUA ≥ 360 μmol/L in women and ≥ 420 μmol/L in men) toward the observed association of variability in SUA with risk of all-cause mortality, we performed a sensitivity analysis excluding individuals with a hyperuricemia on follow-up. Second, given that other common diseases may have additional effects on all-cause mortality, we performed another sensitivity analysis by excluding participants with cardiovascular events (myocardial infarction and stroke) during baseline and follow-up. Third, considering SUA variability may be affected by kidney function, we further adjusted for eGFR variability during 2006–2010 on the basis of model 3. Finally, stratified analyses were performed to evaluate whether the association between SUA variability and all-cause mortality modified by age (< 45, 45–54, 55–64, ≥ 65 years), gender (female vs. male), baseline SUA (< 300 vs. ≥ 300 μmol/L), hypertension (no vs. yes), diabetes mellitus (no vs. yes), dyslipidemia (no vs. yes), BMI (< 25 vs. ≥ 25 kg/m^2^), and eGFR (< 90 vs. ≥ 90 ml/min/1.73m^2^); interactions between subgroups were analyzed using likelihood ratio test comparing models with and those without multiplicative interaction terms.

Furthermore, to investigate the potential mechanisms of variability of SUA on all-cause mortality, we compared the changes in common risk factors related to all-cause mortality over time, including mean values of SBP, BMI, TC, FBG, eGFR, and hs-CRP from baseline through the 4-year follow-up among categories of variability of SUA.

All analyses were conducted using SAS version 9.4 (SAS Institute Inc., Cary, NC, USA). A two-sided *P* < 0.05 was considered statistically significant.

## Result

### Baseline characteristics

The current study included 53,956 participants (mean age, 49.24 ± 11.83 years; men, 76.38%), the mean SUA variability, as measured by CV, was 16.30 ± 10.68%. The baseline characteristics of participants stratified by quartiles of SUA variability are presented in Table 1. The participants with greater variability were younger; were more likely to be men; were less educated; had more current smokers; were drinkers; had more active physical activity; had a higher prevalence of hypertension, diabetes, and dyslipidemia; were more antihypertensive agents takers; and were more common with higher BMI, SBP, DBP, FBG, lower eGFR, and higher hs-CRP, baseline SUA, and mean SUA levels.

**Table 1:** Baseline characteristics of each group categorized by serum uric acid variability (measured by CV)

Characteristics	Total (*N* = 53,956)	Quartile 1 (*N* = 13,483)	Quartile 2 (*N* = 13,495)	Quartile 3 (*N* = 13,492)	Quartile 4 (*N* = 13,486)	*P* value
Age, years	49.24 ± 11.83	49.71 ± 11.30	49.35 ± 11.61	49.05 ± 11.90	48.85 ± 12.45	< 0.0001
Male, *n* (%)	41,210 (76.38)	9790 (72.61)	10,037 (74.38)	10,372 (76.88)	11,011 (81.65)	< 0.0001
High school or above, *n* (%)	4262 (7.9)	1086 (8.06)	1141 (8.46)	1061 (7.86)	974 (7.22)	0.0021
Income ≥ 1000 RMB, *n* (%)	8204 (15.2)	1933 (14.34)	2098 (15.55)	2122 (15.73)	2051 (15.21)	0.0075
Current smoker, *n* (%)	18,307 (33.93)	4204 (31.18)	4340 (32.16)	4640 (34.39)	5123 (37.99)	< 0.0001
Current alcohol, *n* (%)	20,899 (38.73)	4720 (35.01)	5008 (37.11)	5326 (39.48)	5845 (43.34)	< 0.0001
Active physical activity, *n* (%)	7746 (14.36)	1806 (13.39)	1924 (14.26)	2003 (14.85)	2013 (14.93)	0.0009
Hypertension, *n* (%)	6188 (11.47)	1330 (9.86)	1426 (10.57)	1564 (11.59)	1868 (13.85)	< 0.0001
Diabetes mellitus, *n* (%)	1492 (2.77)	377 (2.80)	387 (2.87)	348 (2.58)	380 (2.82)	0.4848
Dyslipidemia, *n* (%)	3428 (6.35)	779 (5.78)	820 (6.08)	866 (6.42)	963 (7.14)	< 0.0001
Antihypertensive agents, *n* (%)	5390 (9.99)	1143 (8.48)	1225 (9.08)	1370 (10.15)	1652 (12.25)	< 0.0001
Hypoglycemic agents, *n* (%)	1153 (2.14)	292 (2.17)	308 (2.28)	267 (1.98)	286 (2.12)	0.3848
Lipid-lowering agents, *n* (%)	543 (1.01)	119 (0.88)	124 (0.92)	140 (1.04)	160 (1.19)	0.0543
Body mass index, kg/m^2^	25.08 ± 3.47	24.87 ± 3.46	24.96 ± 3.48	25.15 ± 3.45	25.36 ± 3.47	< 0.0001
Systolic blood pressure, mmHg	128.61 ± 19.93	127.56 ± 19.55	127.90 ± 19.54	128.48 ± 19.67	130.51 ± 20.80	< 0.0001
Diastolic blood pressure, mmHg	82.68 ± 11.37	81.95 ± 11.09	82.24 ± 11.21	82.63 ± 11.23	83.91 ± 11.84	< 0.0001
Fasting blood glucose, mmol/L	5.40 ± 1.55	5.34 ± 1.54	5.39 ± 1.58	5.39 ± 1.47	5.47 ± 1.61	< 0.0001
eGFR, mL/min/1.73 m^2^	84.06 ± 25.13	86.46 ± 28.23	84.13 ± 24.28	83.20 ± 25.11	82.46 ± 22.38	< 0.0001
hs-CRP, mg/L	2.34 ± 6.50	2.03 ± 5.23	2.11 ± 4.98	2.36 ± 7.45	2.87 ± 7.79	< 0.0001
Serum uric acid, μmol/L						
Baseline	286.36 ± 83.74	272.33 ± 68.36	280.05 ± 71.98	286.99 ± 79.16	306.08 ± 106.33	< 0.0001
Mean	287.99 ± 72.43	272.52 ± 67.26	280.97 ± 68.16	289.62 ± 70.51	308.85 ± 78.23	< 0.0001
Serum uric acid variability						
CV	16.30 ± 10.68	5.92 ± 2.71	11.84 ± 3.26	17.86 ± 4.82	29.58 ± 10.42	< 0.0001
SD	45.75 ± 31.14	15.23 ± 5.76	31.46 ± 4.41	48.87 ± 5.89	87.43 ± 30.16	< 0.0001
ARV	56.26 ± 40.36	19.41 ± 8.90	39.64 ± 11.27	60.67 ± 16.64	105.32 ± 44.43	< 0.0001
VIM	9.07 ± 5.94	3.29 ± 1.51	6.59 ± 1.82	9.94 ± 2.68	16.47 ± 5.80	< 0.0001

*eGFR* estimated glomerular filtration rate, *hs-CRP* high-sensitivity C-reactive protein, *CV* coefficient of variation, *SD* standard deviation, *ARV* average real variability, *VIM* variability independent of the mean

Continuous variables are expressed as mean with standard deviation; categorical variables are expressed as frequency with percentage

There was a linear association between baseline SUA and all-cause mortality; the inflection point we detected was 300 μmol/L; when baseline SUA was over 300 μmol/L, per 60 μmol/L increased was linked to 8% higher risk of all-cause mortality (hazard ratio (HR), 1.08; 95% CI, 1.03–1.13) (SFigure 3, see Additional file 3).

### Variability in SUA and risk of all-cause mortality

**Fig. 1:**
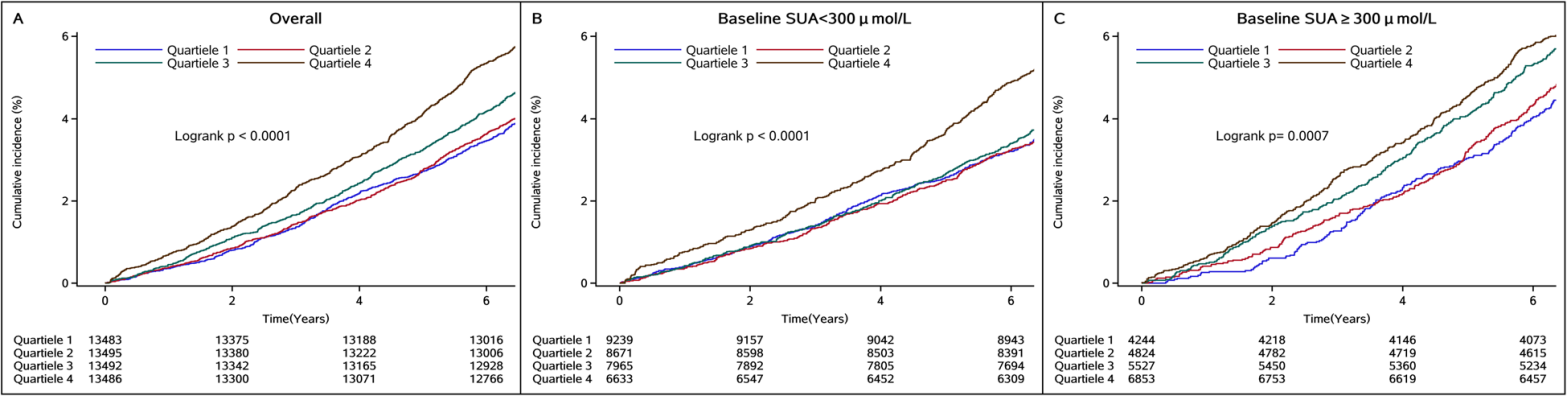
Kaplan-Meier curve of all-cause mortality incidence rate by variability in serum uric acid. Abbreviations: SUA, serum uric acid

During a median follow-up of 7.04 (interquartile range 6.67–7.31) years, 2728 subjects died. The incidence rate of all-cause mortality increased from 6.71 in the lowest quartile to 8.19 per 1000 person-years in the highest quartile of SUA variability. Kaplan-Meier also showed that individuals with the highest variability of SUA experienced the higher risk of all-cause mortality than other participants during the 7.04-year follow-up (log-rank test, *P* < 0.0001, Fig. 1a–c).

**Fig. 2:**
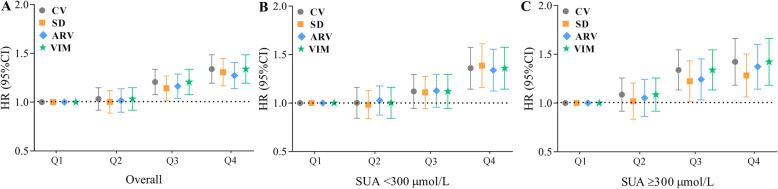
Hazard ratios and 95% confidence intervals of the associations between various variability measurements of serum uric acid and risk of all-cause mortality. ARV, average real variability; CV, coefficient variability; CI, confidence interval; HR, hazard ratio; SD, standard deviation; SUA, serum uric acid; VIM, variability independent of the mean. Adjusted for age and gender, body mass index, systolic blood pressure, diastolic blood pressure, fasting blood glucose, education, income, smoking status, drinking status, physical activity, history of hypertension, diabetes and dyslipidemia, antihypertensive agents, hypoglycemic agents, lipid-lowering agents, estimated glomerular filtration rate, high-sensitivity C-reactive protein, baseline serum uric acid, and mean serum uric acid

In the fully adjusted model, participants with the highest variability of SUA had higher risk of all-cause mortality in the overall cohort as well as different baseline SUA categories, the HRs were 1.33 (95% CI, 1.20–1.49; *P* for trend < 0.0001) for the overall, 1.38 (95% CI, 1.18–1.61; *P* for trend < 0.0001) for baseline SUA < 300, and 1.42 (95% CI 1.20–1.60; *P* for trend < 0.0001) for baseline SUA ≥ 300 μmol/L (Table 2). The association persisted when SUA variability measured using SD, ARV, or VIM (Fig. 2a–c). Sensitivity analyses demonstrated similar results when individuals with a hyperuricemia event on follow-up were removed (*n* = 8429), when the analysis was restricted to those without cardiovascular and cerebrovascular diseases from baseline through follow-up (*n* = 3594), and when we further adjusted for eGFR variability (Table 2).

**Table 2:** Hazard ratios and 95% confidence intervals of all-cause mortality by quartiles of serum uric acid variability (measured by CV)

Variable	Quartile 1	Quartile 2	Quartile 3	Quartile 4	Per-SD increase	*P*_trend_
Overall						
No. of cases	592 (4.29)	606 (4.49)	689 (5.11)	841 (6.24)		
Incidence rate^a^	6.71 (6.20–7.26)	6.84 (6.33–7.40)	7.81 (7.26–8.40)	8.19 (7.63–8.80)		
Model 1	Reference	1.03 (0.93–1.16)	1.22 (1.10–1.36)	1.40 (1.26–1.56)	1.14 (1.10–1.18)	< 0.0001
Model 2	Reference	1.03 (0.92–1.15)	1.20 (1.08–1.34)	1.34 (1.20–1.49)	1.12 (1.08–1.16)	< 0.0001
Model 3	Reference	1.03 (0.92–1.15)	1.20 (1.08–1.34)	1.33 (1.20–1.49)	1.12 (1.08–1.15)	< 0.0001
Sensitivity analysis^b^	Reference	1.00 (0.88–1.13)	1.15 (1.02–1.30)	1.25 (1.10–1.42)	1.10 (1.05–1.15)	< 0.0001
Sensitivity analysis^c^	Reference	1.01 (0.89–1.14)	1.16 (1.03–1.31)	1.33 (1.18–1.49)	1.12 (1.07–1.16)	< 0.0001
Sensitivity analysis^d^	Reference	1.03 (0.92–1.15)	1.19 (1.06–1.32)	1.30 (1.07–1.45)	1.10 (1.06–1.15)	< 0.0001
Baseline SUA < 300 μmol/L						
No. of cases	301 (4.14)	311 (3.93)	371 (4.32)	450 (5.14)		
Incidence rate^a^	6.02 (5.38–6.74)	5.71 (5.11–6.38)	6.29 (5.68–6.96)	7.51 (6.84–8.23)		
Model 1	Reference	1.00 (0.85–1.17)	1.11 (0.96–1.30)	1.34 (1.16–1.55)	1.13 (1.07–1.19)	< 0.0001
Model 2	Reference	1.00 (0.85–1.17)	1.10 (0.95–1.29)	1.33 (1.14–1.54)	1.15 (1.08–1.22)	< 0.0001
Model 3	Reference	1.01 (0.86–1.19)	1.13 (0.97–1.31)	1.38 (1.18–1.61)	1.15 (1.08–1.21)	< 0.0001
Sensitivity analysis^b^	Reference	1.01 (0.86–1.18)	1.12 (0.96–1.31)	1.31 (1.11–1.54)	1.13 (1.06–1.20)	< 0.0001
Sensitivity analysis^c^	Reference	1.01 (0.89–1.14)	1.16 (1.03–1.31)	1.33 (1.18–1.49)	1.15 (1.08–1.22)	< 0.0001
Sensitivity analysis^d^	Reference	0.99 (0.84–1.16)	1.10 (0.94–1.28)	1.31 (1.12–1.53)	1.13 (1.07–1.20)	< 0.0001
Baseline SUA ≥ 300 μmol/L	Reference	1.01 (0.85–1.20)	1.12 (0.95–1.32)	1.35 (1.14–1.60)	1.12 (1.06–1.20)	< 0.0001
No. of cases	319 (5.12)	321 (5.75)	349 (7.12)	306 (6.45)		
Incidence rate^a^	7.52 (6.74–8.39)	8.48 (7.60–9.46)	10.50 (9.49–11.70)	9.47 (8.46–10.60)		
Model 1	Reference	1.07 (0.92–1.25)	1.36 (1.17–1.58)	1.48 (1.26–1.73)	1.14 (1.09–1.21)	< 0.0001
Model 2	Reference	1.07 (0.92–1.25)	1.32 (1.14–1.54)	1.42 (1.21–1.67)	1.13 (1.06–1.20)	< 0.0001
Model 3	Reference	1.08 (0.92–1.26)	1.32 (1.13–1.54)	1.42 (1.20–1.69)	1.12 (1.06–1.20)	< 0.0001
Sensitivity analysis^b^	Reference	1.04 (0.85–1.26)	1.42 (1.15–1.76)	1.64 (1.22–2.19)	1.18 (1.05–1.32)	< 0.0001
Sensitivity analysis^c^	Reference	1.02 (0.85–1.21)	1.24 (1.04–1.47)	1.42 (1.17–1.72)	1.13 (1.05–1.21)	< 0.0001
Sensitivity analysis^d^	Reference	1.07 (0.92–1.25)	1.32 (1.13–1.54)	1.38 (1.16–1.64)	1.12 (1.05–1.19)	< 0.0001

*CV* coefficient of variation, *SD* standard deviation

Model 1 adjusted for age and gender

Model 2 further adjusted for body mass index, systolic blood pressure, diastolic blood pressure, fasting blood glucose, education, income, smoking status, drinking status, physical activity, history of hypertension, diabetes, and dyslipidemia

Model 3 further adjusted for antihypertensive agents, hypoglycemic agents, lipid-lowering agents, estimated glomerular filtration rate, high-sensitivity C-reactive protein, baseline serum uric acid, and mean serum uric acid

^a^Incidence rate per 1000 person-years

^b^Sensitivity analysis was excluded the participants with a hyperuricemia event at baseline or in the follow-up period, and adjusted for variables in model 3

^c^Sensitivity analysis was excluded the participants with myocardial infarction or stroke event at baseline or in the follow-up period, and adjusted for variables in model 3

^d^Sensitivity analysis was adjusted for variables in model 3 plus variability of estimated glomerular filtration rate during 2006–2010

**Fig. 3:**
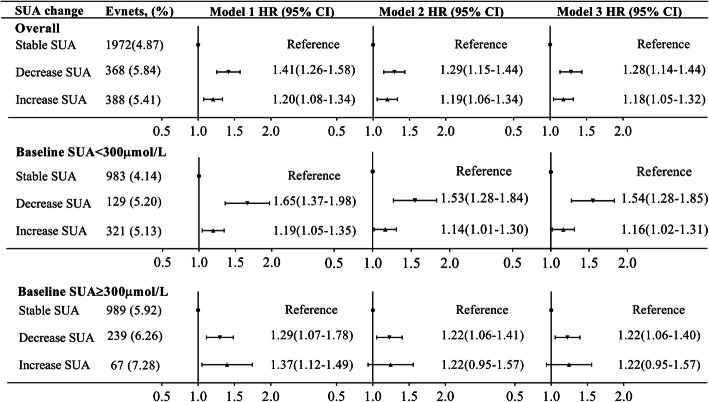
Fall or rise in SUA and risk of all-cause mortality. SUA, serum uric acid; HR, hazard ratio; CI, confidence interval. Model 1 adjusted for age and gender. Model 2 further adjusted for body mass index, systolic blood pressure, diastolic blood pressure, fasting blood glucose, education, income, smoking status, drinking status, physical activity, history of hypertension, diabetes, and dyslipidemia. Model 3 further adjusted for antihypertensive agents, hypoglycemic agents, lipid-lowering agents, estimated glomerular filtration rate, high-sensitivity C-reactive protein, baseline serum uric acid, and mean serum uric acid

Moreover, in the highest variability group, both a large fall (HR, 1.28; 95% CI, 1.14–1.44) and rise (HR, 1.18; 95% CI, 1.05–1.32) in SUA were related to elevated risk of all-cause mortality. A large fall of SUA was significantly associated with risk of all-cause mortality for different baseline categories; however, the relationship of a large rise of SUA and all-cause mortality was significant only for those with baseline SUA < 300 μmol/L (HR, 1.54; 95% CI, 1.28–1.85; Fig. 3).

### Subgroup analysis

Subgroup analyses indicated that the higher risk of all-cause mortality among participants with higher variability of SUA was consistent across relevant subgroups (*P* for interaction > 0.05 for all), including age (< 45, 45–54, 55–64, ≥ 65 years), gender (female vs. male), baseline SUA (< 300 vs. ≥ 300 μmol/L), hypertension (no vs. yes), diabetes mellitus (no vs. yes), dyslipidemia (no vs. yes), BMI (< 25 vs. ≥ 25 kg/m^2^), and eGFR (< 90 vs. ≥ 90 ml/min/1.73m^2^), indicating no significant effect modification of the association between variability of SUA and all-cause mortality (STable 1, see Additional file 4).

### Changes in risk factors related to all-cause mortality

**Fig. 4:**
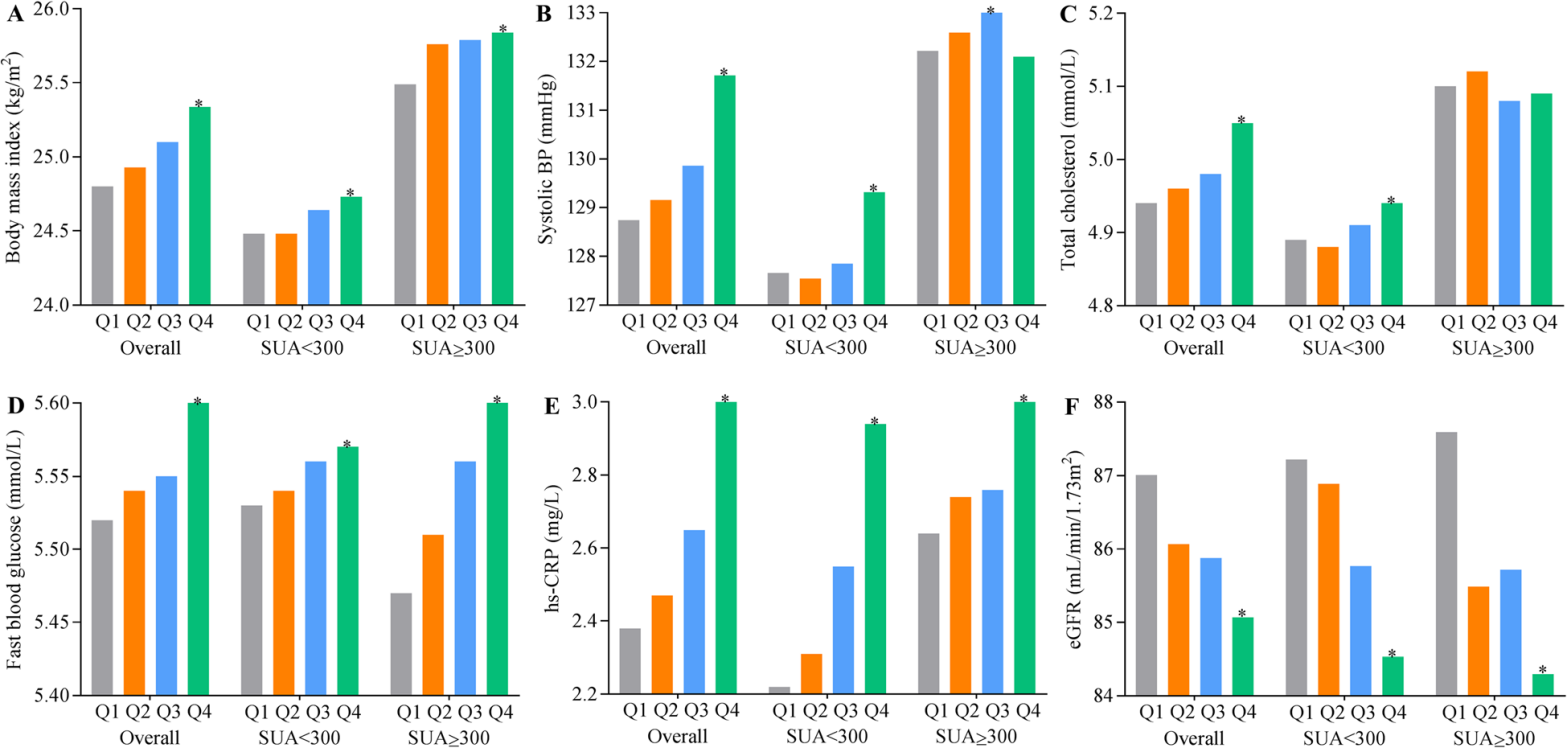
Mean values of risk factors associated with all-cause mortality according to SUA variability. BP, blood pressure; eGFR, estimated glomerular filtration rate; hs-CRP, high-sensitivity C-reactive protein; SUA, serum uric acid. **a** Body mass index. **b** Systolic blood pressure. **c** Total cholesterol. **d** Fasting blood glucose. **e** hs-CRP. **f** eGFR

Figure 4 shows the mean values of BMI, SBP, TC, FBG, eGFR, and hs-CRP from baseline through 2010, according to variability of SUA. The highest SUA variability was significantly related to higher levels of BMI, SBP, TC, FBG, hs-CRP, and lower eGFR level than the stable SUA. Consistent results were observed for different baseline categories, except there was no significant difference in mean value of TC levels for those with baseline SUA ≥ 300 μmol/L.

## Discussion

In this large community-based cohort study, we found that higher long-term variability of SUA was significantly associated with elevated risk of all-cause mortality in the overall cohort and across different baseline SUA categories. The associations were similar for a large fall and rise of SUA. Furthermore, those with higher variability of SUA were relate to higher mean BMI, SBP, TC, FBG, and hs-CRP and lower eGFR levels compared with stable SUA over time. These findings highlight the significance of SUA variability in forecasting future risk of all-cause mortality in general population.

Some prior studies revealed that higher baseline SUA were linked to increased risk of all-cause mortality [8–10]. On the contrary, some studies reported an inverse relationship between baseline SUA and all-cause mortality [11, 12]. Furthermore, recent studies suggested a U-shaped association between baseline SUA and all-cause mortality [13, 14]. These inconsistencies may be due to SUA was only measured once at baseline. Variability takes a fundamental role in all the main control systems of our body, it seems mandatory for some biological parameters to maintain in a very strict narrow range; otherwise, it will be harmful for the body. For example, higher variability in blood pressure, glucose level, cholesterol level, and body weight was independently associated with a wide range of adverse health outcomes, such as cardiovascular events, diabetes, end-stage renal disease, dementia, and all-cause mortality [15–18, 29]. However, the relationship between long-term variability of SUA and risk of all-cause mortality in the general population was less well established until now.

A cohort study included 3202 patients who received successful coronary intervention and with at least three SUA measurements from 2006 to 2015 suggested that subjects in the highest quartile SD group had a higher risk of major adverse cardiovascular events, myocardial infraction, cardiovascular death, heart failure-related hospitalization, and total major CV events during an average follow-up of 65.06 months [19]. Additionally, another study with 10,059 male tenured civil servants and municipal employees in Israel showed a higher SUA variability at midlife was an independent predictor of coronary heart disease mortality and all-cause mortality [20].

Consistent with these studies, the association of variability of SUA and outcomes was extent to the general population in our current study by demonstrating a robust, significant association between various measurements of SUA variability and risk of all-cause mortality; the associations were similar for both a large fall and rise of SUA in the highest variability group. Moreover, these associations were consistent across different baseline SUA levels, although the association between a large rise of SUA and all-cause mortality did not reach a significant level, which may due to the small size (67 cases) of outcome of interest in this group.

Although the variability of SUA may be affected by administration of SUA-lowering therapies, a meta-analysis of 35 randomized controlled trials in patients with gout showed that UA-lowering therapy did not reduced the composite of CVD death, non-fatal myocardial infarction, or non-fatal stroke or all-cause mortality compared with the placebo [30]. Since the information on UA-lowering therapies was not available in our study, the question that whether variability of SUA is a risk factor or simply a correlate (epiphenomenon) of all-cause mortality risk clustered in subjects with elevated variation of SUA may needed further investigations to answer.

In addition, we also found participants with higher variability of SUA had higher BMI, SBP, TC, FBG, hs-CRP, and lower eGFR levels than those with more stable SUA over time; these parameters may present cardiac metabolism, systematic inflammation, and renal function and have an important impact on the pathophysiology of mortality [15–18, 31, 32]. The finding was in agreement with the study of Ceriello et al., which investigated variability in glycated hemoglobin, blood pressure, lipids, and SUA with the risk of chronic kidney disease in type 2 diabetes, and reported that high variability in all the aforementioned parameters predicted the decline in eGFR, among which high variability in SUA conferred the highest risk [21]. The relationship between variability of SUA and these parameters may provide a potential pathway by which SUA variability may affect risk of all-cause mortality.

Although potential biologically mechanisms underlying the association between variability in SUA and risk of all-cause mortality are not well established, several hypotheses have been post. First, SUA level could be assumed as a marker of metabolic changes and was related to endothelial dysfunction, oxidative stress, and the magnitude of activation of the renin-angiotensin system; then, SUA fluctuations may accelerate these important issues and thus contribute to the progression of mortality [33, 34]. Second, a surge of UA in the blood is known to increase the crystallization rate of urate, which stimulates an immune reaction and inflammatory response [35]. Third, individuals with a high SUA variability usually have some coexisting mortality risk factors (e.g., smoking, drinking, hypertension, diabetes, dyslipidemia, antihypertensive agents), indicating a high SUA variability may reflect patients with more risk factors in some way. Another plausible mechanism could be increased incidence of hyperuricemia events among individuals with higher variability of SUA, which has been demonstrated to be associated with risk of all-cause mortality strongly in prior studies [8, 36, 37]. Further research is still required to investigate the clear mechanism for the association.

The strengths of our study include the prospective design, large population, and use different measurements of variability of SUA to robust the findings. However, our study has several limitations. First, the information on urate-lowering therapies was not recorded in our study, which may have a potential effect on the association between variability of SUA and the risk of all-cause mortality. Second, we measured SUA within the first three waves and did not investigate long-term variability in SUA value. This design was chosen in order to maximize the number of participants with SUA measurements before 2010 and to allow a longer follow-up period to capture the occurrence of death. Third, we did not collect information on specific causes of death, but we used sensitivity analysis by excluding participants who suffered cardiovascular diseases. Finally, owing to the observational nature of the study, we cannot establish a causal association between SUA variability and risk of all-cause mortality; thus, our findings need to be confirmed in future studies. Furthermore, we cannot exclude the possibility of residual or unmeasured confounding given the observational study design of the present analysis.

## Conclusions

In conclusion, our finding suggest higher visit-to visit variability in SUA are associated with an increased risk for all-cause mortality irrespective of baseline SUA level and the direction of variability. Our study highlights the importance of achieving stable SUA levels and avoiding large fluctuations and may help to determine the real high-risk population and design future studies for therapy.

## Supplementary Information

**Additional file 1: Figure S1.** Time line of the study.

**Additional file 2: Figure S2.** Flowchart of the study.

**Additional file 3: Figure S3.** Multivariable-adjusted hazard ratio and 95% confidence interval for baseline SUA and all-cause mortality.

**Additional file 4: Table S1.** Subgroup analyses for the association of serum uric acid variability and all-cause mortality.

## Data Availability

The data generated by our research could be made available upon request to the corresponding authors.

## Abbreviations

ARV: Average real variability
BMI: Body mass index
CI: Confidence interval
CV: Coefficient of variation
DBP: Diastolic blood pressure
eGFR: Estimated glomerular filtration rate
HR: Hazard ratio
hs-CRP: High-sensitivity C-reactive protein
SBP: Systolic blood pressure
SD: Standard deviation
SUA: Serum uric acid
TC: Total cholesterol
VIM: Variability independent of the mean
