# Supplementary material for: Visit-to-visit variability of serum uric acid measurements and the risk of all-cause mortality in the general population
Source: Arthritis Res Ther. 2021 Mar 4;23:74. doi: 10.1186/s13075-021-02445-7 (PMC7931538; doi:10.1186/s13075-021-02445-7)
Supplement: Supplementary file 1 — Additional file 1: Figure S1. Time line of the study. [file 13075_2021_2445_MOESM1_ESM.docx]

Year 2006

Year 2008

Year

2010

Serum uric acid assessment period

Follow-up for outcomes ascertainment

End of follow-up

(December 2017)

Figure S1. Time line of the study
