# Supplementary material for: Visit-to-visit variability of serum uric acid measurements and the risk of all-cause mortality in the general population
Source: Arthritis Res Ther. 2021 Mar 4;23:74. doi: 10.1186/s13075-021-02445-7 (PMC7931538; doi:10.1186/s13075-021-02445-7)
Supplement: Supplementary file 2 — Additional file 2: Figure S2. Flowchart of the study. [file 13075_2021_2445_MOESM2_ESM.docx]

Figure S2. Flowchart of the study
