# Supplementary material for: Visit-to-visit variability of serum uric acid measurements and the risk of all-cause mortality in the general population
Source: Arthritis Res Ther. 2021 Mar 4;23:74. doi: 10.1186/s13075-021-02445-7 (PMC7931538; doi:10.1186/s13075-021-02445-7)
Supplement: Supplementary file 3 — Additional file 3: Figure S3. Multivariable-adjusted hazard ratio and 95% confidence interval for baseline SUA and all-cause mortality. [file 13075_2021_2445_MOESM3_ESM.docx]

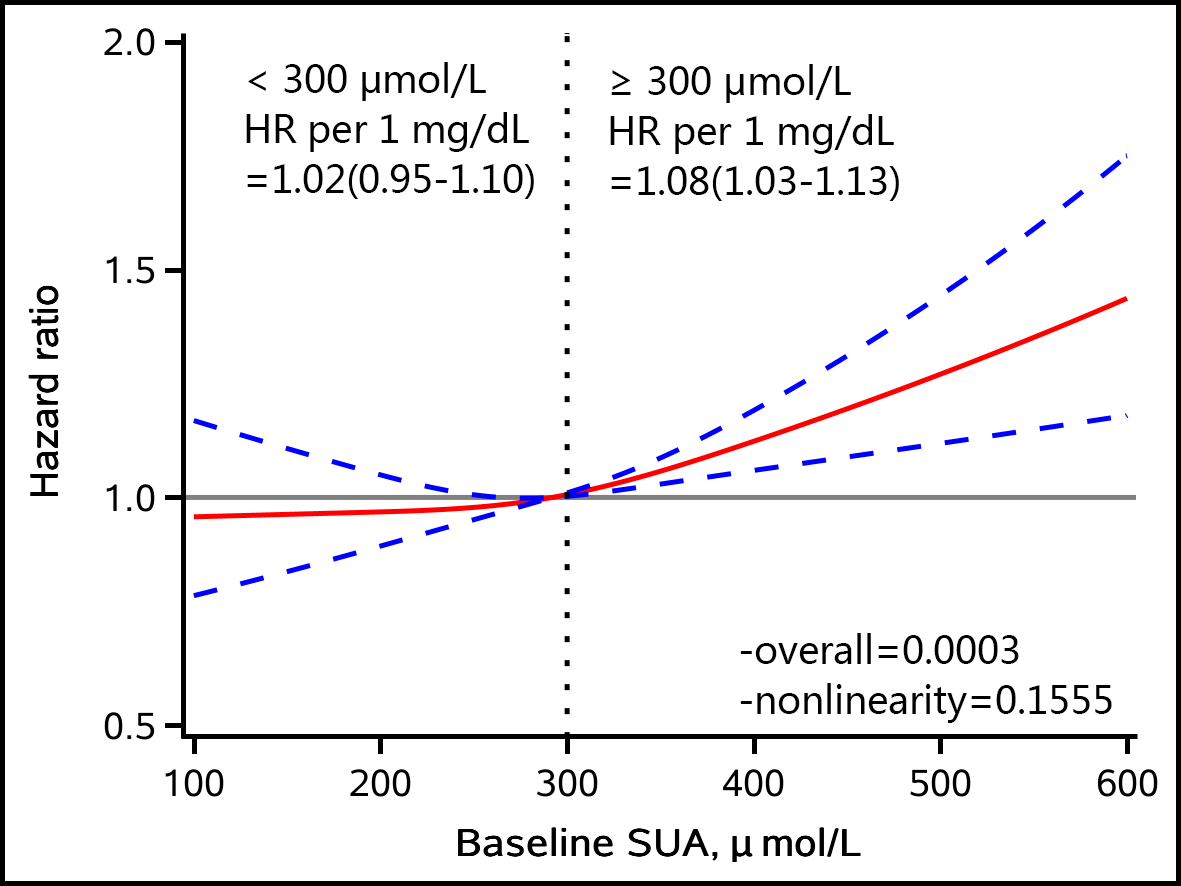


**Figure S3. Multivariable-adjusted hazard ratio and 95% confidence interval for baseline SUA and all-cause mortality .**

Abbreviation: SUA, serum uric acid.

Red line represented adjusted hazard ratio (with 95% confidence limits [dashed lines]) based on restricted cubic splines with knots at the 5^th^, 25^th^, 50^th^, 75^th^, and 95^th^ percentiles of baseline serum uric acid distribution.

Adjusted for age and gender, body mass index, systolic blood pressure, diastolic blood pressure, fasting blood glucose, education, income, smoking status, drinking status, physical activity, history of hypertension, diabetes and dyslipidemia, antihypertensive agents, hypoglycemic agents, lipid-lowering agents, estimated glomerular filtration rate, C-reactive protein.
