# Supplementary material for: Visit-to-visit variability of serum uric acid measurements and the risk of all-cause mortality in the general population
Source: Arthritis Res Ther. 2021 Mar 4;23:74. doi: 10.1186/s13075-021-02445-7 (PMC7931538; doi:10.1186/s13075-021-02445-7)
Supplement: Supplementary file 4 — Additional file 4: Table S1. Subgroup analyses for the association of serum uric acid variability and all-cause mortality. [file 13075_2021_2445_MOESM4_ESM.docx]

Table S1. Subgroup analyses for the association of serum uric acid variability and all-cause mortality

| Variables | Group | Model 3 | *P* for interaction |
| --- | --- | --- | --- |
| Age |  |  |  |
| < 60 years | Quartile 4 | 1.30(1.11-1.52) | 0.5993 |
| ≥ 60 years | Quartile 4 | 1.34(1.16-1.56) |  |
| Gender |  |  |  |
| Female | Quartile 4 | 1.42(1.01-1.99) | 0.9282 |
| Male | Quartile 4 | 1.31(1.17-1.47) |  |
| Baseline SUA |  |  |  |
| < 300 μmol/L | Quartile 4 | 1.35(1.15-1.58) | 0.3839 |
| ≥ 300 μmol/L | Quartile 4 | 1.38(1.16-1.64) |  |
| Hypertension |  |  |  |
| No | Quartile 4 | 1.32(1.16-1.49) | 0.9110 |
| Yes | Quartile 4 | 1.37(1.09-1.71) |  |
| Diabetes mellitus |  |  |  |
| No | Quartile 4 | 1.34(1.20-1.49) | 0.6996 |
| Yes | Quartile 4 | 1.19(0.79-1.80) |  |
| Dyslipidemia |  |  |  |
| No | Quartile 4 | 1.32(1.18-1.47) | 0.8059 |
| Yes | Quartile 4 | 1.46(1.03-2.06) |  |
| eGFR |  |  |  |
| <90 mL/min/1.73m^2^ | Quartile 4 | 1.33(1.18-1.50) | 0.3363 |
| ≥ 90 mL/min/1.73m^2^ | Quartile 4 | 1.33(1.03-1.71) |  |
| BMI |  |  |  |
| < 25 kg/m^2^ | Quartile 4 | 1.42(1.22-1.66) | 0.4847 |
| ≥ 25 kg/m^2^ | Quartile 4 | 1.24(1.07-1.44) |  |

Abbreviations: SUA, serum uric acid; eGFR, ; BMI, body mass index.

Adjusted for age and gender, body mass index, systolic blood pressure, diastolic blood pressure, fasting blood glucose, education, income, smoking status, drinking status, physical activity, history of hypertension, diabetes and dyslipidemia, antihypertensive agents, hypoglycemic agents, lipid-lowering agents, estimated glomerular filtration rate, high-sensitivity C-reactive protein, baseline serum uric acid, and mean serum uric acid.
